# Association between Tumor Necrosis Factor-α rs1800629 Polymorphism and Risk of Asthma: A Meta-Analysis

**DOI:** 10.1371/journal.pone.0099962

**Published:** 2014-06-17

**Authors:** Guangdie Yang, Junjun Chen, Fei Xu, Zhang Bao, Yake Yao, Jianying Zhou

**Affiliations:** Department of Respiratory Diseases, The First Affiliated Hospital of College of Medicine, Zhejiang University, Hangzhou, Zhejiang, People’s Republic of China; Rutgers - New Jersey Medical School, United States of America

## Abstract

**Objective:**

The purpose of this study was to explore the association between the TNF-α rs1800629 (also refers as -308G/A) polymorphism and asthma susceptibility.

**Methods:**

We searched the Pubmed, Embase, Cochrane Central Register of Controlled Trials (CENTRAL) and Wanfang databases. Odds ratios (ORs) with 95% confidence intervals (CIs) were used to calculate the strength of association.

**Results:**

A total of 34 studies involving 5477 asthma patients and 5962 controls were included in present study. The results indicated that TNF-α rs1800629 polymorphism was significantly associated with asthma risk in a recessive genetic model (OR = 1.46, 95% CI 1.21–1.76, P<0.0001). Subgroup analyses found that the TNF-α rs1800629 polymorphism was significantly associated with asthma risk in West Asians and South Asians (OR = 2.47, 95% CI = 1.48–4.12, P = 0.0005; OR = 1.83, 95% CI = 1.42–2.36, P<0.00001), but not East Asians and Caucasians. Furthermore, significant association also was observed in allergic asthma (OR = 1.51, 95% CI = 1.24–1.83, P<0.0001), adults and children (OR = 1.43, 95 CI% = 1.07–1.91, P = 0.02; OR = 1.57, 95% CI = 1.19–2.06, P = 0.001).

**Conclusions:**

This meta-analysis suggested that the rs1800629 polymorphism in TNF-α was a risk factor for asthma.

## Introduction

Asthma is a chronic allergic disorder of the airways that is characterized by inflammatory infiltrates in the bronchial walls, airway hyperresponsiveness (AHR) and reversible airway obstruction [1]. It is believed to be a multifactorial disease whereby genetic factors contribute to its etiology [2]. To date, numerous risk genes have been reported to be associated with asthma susceptibility in various populations [3]. One of the candidate susceptible genes which has been intensely investigated is tumor necrosis factor-alpha (TNF-α).

TNF-α is a proinflammatory cytokine released during allergic responses by both macrophages and mast cells [4]. Sharma et al. reported that TNF-α levels in the sputum and bronchoalveolar lavage fluid of asthmatic patients were increased [5]. Furthermore, a meta-analysis performed by Chen et al. demonstrated that TNF-α antagonism therapy was superior to control therapy in preventing exacerbations in patients with asthma [6]. These accumulated data support the idea that TNF-α plays an important role in the pathogenesis of asthma and the TNF-α gene may be a susceptibility gene of asthma.

TNF-α gene is located on chromosome 6p21 within the major histocompatibility complex class III region [7], which has shown the evidence of linkage to asthma, atopy, and related phenotypes in multiple genome-wide studies [8], [9]. Up to now, a lot of studies of genetic epidemiology have assessed the association of TNF-α gene polymorphisms and risk of asthma in different populations [5], [10]–[40]. Most of them focused on rs1800629 (also referred to as -308G/A). However, the results from these studies were often inconsistent and inconclusive. As early as 2000, Lois et al. [10] published their negative findings regarding this issue, and their results were supported by other researchers, including Buckova et al. [12] and Kamali et al. [25]. In contrast, Guo et al. [16] showed that the TNF-alpha rs1800629 polymorphism was strongly associated with the risk of asthma, which was consistent with the results of Gupta et al. [22] and Jiffri et al. [35]. Although several meta-analyses on this polymorphism have been published [41]–[43], some conflicting results still existed and were needed to be further investigated. For instance, Gao J et al. did not discuss the age-specific effects of this polymorphism on asthma risk, and also neglected atopic status and ethnic status [41]. Aoki et al. [42] found that TNF-α-308A allele was associated with an increased risk of asthma among Asians and Caucasians. However, Zhang et al. [43] showed that significant elevated risks were associated with A allele carriers in Asians but not in Caucasians. In addition, Zhang et al. [43] focused on published studies written in English and Chinese which may influence the results of genetic effects. Furthermore, there is new methodoloical development in meta-analyses adopted the classical method having the limitation of increasing type I error because of involving multicomparison [44], [45]. Therefore, we performed a meta-analysis of all eligible studies to obtain more precise estimation of the association of TNF-α rs1800629 polymorphism with asthma susceptibility.

## Methods

### Publication Search

We conducted an elaborate search for studies that examined the association of TNF-α polymorphisms with asthma. Two independent reviewers (Chen and Bao) searched PUBMED, EMBASE, Cochrane Central Register of Controlled Trials (CENTRAL) and Wanfang databases to identify available studies published up to November 2013. The heading (MeSH) terms and/or text words which were used were as follows: ‘asthma or asthmatic or bronchial spasm or respiratory hypersensitivity’, and in combination with ‘tumor necrosis factor or TNF or tumor necrosis factor-α or TNF-α or TNF-alpha or tumor necrosis factor-alpha’, and in combination with ‘polymorphism or variant or genetic or SNP’. We also perused the reference lists of all retrieved articles and relevant reviews. There are no restrictions were placed on language, race, ethnicity or geographic area.

### Study Selection and Data Extraction

Studies were included in this meta-analysis if meeting the following criteria: (1) evaluation of the polymorphisms in TNF-α gene and asthma risk; (2) they were case-control studies; (3) genotype distributions in both asthmatics and controls should be sufficient to calculate an odds ratio (OR) with 95% confidence interval (CI). The studies were excluded if (1) studies containing overlapping data; (2) studies based on family; (3) studies in which the number of null and wild genotypes or alleles were not offered; (4) editorials, reviews and abstracts; (5) studies which were not consistent with Hardy-Weinberg equilibrium (HWE).

Data were extracted from original studies independently by two reviewers (Chen and Xu). Discrepancy between the reviewers was resolved by consensus or a third reviewer (Yao). The following information was collected from each study: first author’s name, year of publication, original country, ethnicity, age group, atopic status, definition of asthma and control patients, genotyping method, the number of cases and controls, and genotype and allele frequency information. We verified accuracy of data by comparing collection forms from each investigator.

### Quality Score Assessment

The quality of studies was assessed by the same two reviewers (Chen and Xu) independently. The quality scoring system (Table S1) was based on both traditional epidemiological considerations and asthma genetic issues recommended by Thakkinstian et al. [46]. Total scores ranged from 0 (worst) to 15 (best). Any disagreement was adjudicated by a third reviewer (Bao). Studies with quality scores ≤4 were considered as low quality studies and excluded from our study [47].

### Statistical Analysis

When the data from at least 5 similar studies were available, meta-analysis was performed. Only one polymorphism (rs1800629) were finally chosen, other polymorphisms such as rs361525 (TNF-238G/A), rs1799724 (TNF-857C/T), rs1799964 (TNF-1031T/C) and rs1800630 (TNF-863C/A) were excluded because of insufficient of studies on each polymorphism. The summary odds ratios (ORs) and 95% confidence intervals (CIs) were used to measure the strength of association between the TNF-α polymorphisms and asthma susceptibility. The statistical significance of summary ORs were evaluated with the Z test. The overall statistical analysis process was performed according to the standard procedure recommended by Thakkinstian et al. [45]. Briefly, 1) the allele comparison was conducted to determine the risk allele; 2) OR1, OR2, and OR3 were explored for the genotypes: GG vs. AA (OR1), GA vs. AA (OR2), and GG vs. GA (OR3) for the rs1800629 polymorphism. These pairwise differences were used to indicate the most appropriate genetic model; 3) after the best genetic effect model was confirmed, this model was used to collapse the three genotypes into two groups (except in the case of a codominant model) and to pool the results.

Heterogeneity among studies was assessed by using the chi-square based Cochrane Q-test, which was considered to be significant for P<0.10, and the inconsistency index (*I^2^*). *I^2^* values of 25, 50, and 75 were normally considered low, moderate, and high heterogeneity [48]. The fixed effects model assumes that a genetic factor which has a similar effect on asthma susceptibility across all studies are caused by chance alone [49]. Alternatively, the random effects model assumes that different studies shows substantial diversity and assesses both within-study sampling errors and between-study variances [50]. The fixed-effect model was adopted when the effects were assumed to be homogeneous, otherwise the random-effect model was used. To explore the source of the heterogeneity and evaluate the ethnicity-specific, atopy-specific, and age-specific effects, subgroup analyses were carried out for the rs1800629 polymorphism, which was investigated in a sufficient number of studies. Departure from HWE in control group was evaluated by the chi-square test.

In order to assess the stability of the results, we did sensitivity analysis by sequentially excluding each study. Publication bias was appraised by visual inspection of funnel plots, in which the standard error of log (OR) of each study was plotted against its log (OR). Funnel plot asymmetry was assessed by Egger’s linear regression test [51].

All statistical tests were calculated by using Review manager 5.2 software (Nordic Cochrane Center, Copenhagen, Denmark) and STATA 12.0 software (Stata Corporation, College Station, TX). A P value <0.05 was considered statistically significant.

## Results

### Included Studies Characteristics

**Figure 1:**
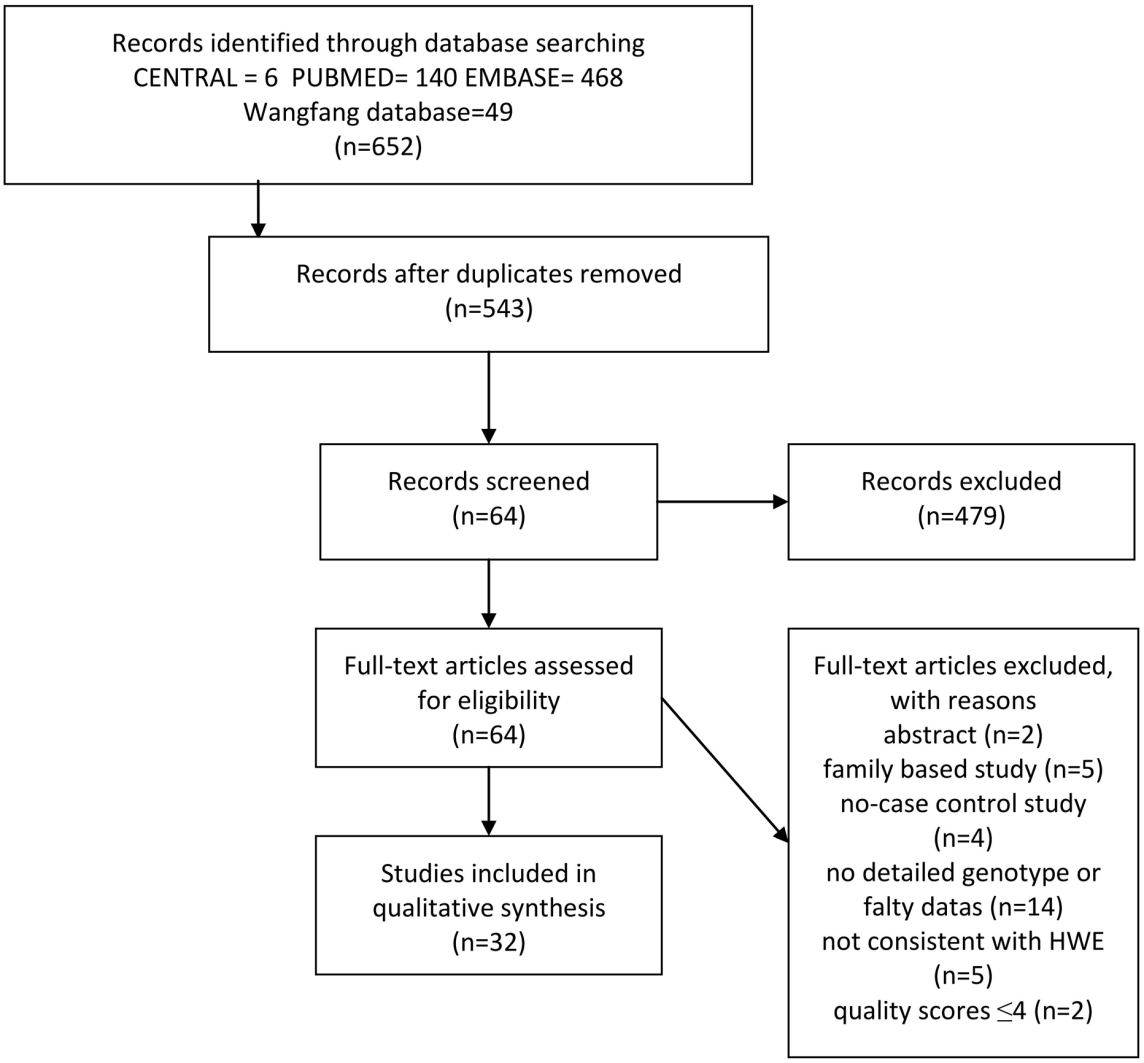
Study flow chart of identification, inclusion, and exclusion.

The flow diagram in Figure 1 summarizes selection process of this literature review. Eventually, a total of 32 eligible articles completely met the inclusion criteria were incorporated into this meta-analysis [5], [10]–[29], [31]–[33], [35]–[40]. Two articles reported two cohorts, and each cohort was considered as a case-control study [5], [11]. At last, there were 34 case-control studies in 32 articles concerning association between the TNF-α rs1800629 polymorphism and asthma risk. There were seven studies performed in Caucasians, 24 studies in Asians. 14 studies involved children alone, 11 recruited adults and six included both children and adults. Four studies included only atopic asthma patients, 12 studies included both of atopic and non-atopic asthma patients, but only three studies of these can be extracted separately. The characteristics of these studies are shown in Table 1. The detailed genotype and frequencies and HWE examination results are listed in Table 2.

**Table 1:** Characteristics of the case-control studies included in meta-analysis.

Study[41]	Year	Country	Ethnicity	Age group	Atopic	Case	Control	Genotyping	Quality
					status	(n)	(n)	method	score
Louis(10)	2000	Belgium	Caucasian	Adult	Mix	95	98	Primer specific	11
								PCR	
Winchester(C)(11)	2000	UK	Caucasian	Children	NA	20	416	RELF	7
Winchester(A)(11)	2000	UK	SA	Children	NA	6	275	RELF	7
Buckova(12)	2002	Czech	Caucasian	Mix	Atopic	151	155	PCR-RFLP	10
Witte(13)	2002	USA	Mix	Adult	NA	235	273	TaqMan assay	9
Gao JM(14)	2003	China	EA	Adult	NA	125	96	PCR-RFLP	7
Beghe(15)	2004	Italy	Caucasian	Adult	Mix	142	45	ARMS-PCR	7
Guo YL(16)	2004	China	EA	Adult	NA	48	21	RFLP	6
Liu DF(17)	2004	China	EA	Children	NA	113	126	PCR-RFLP	6
Shin(18)	2004	Korea	EA	Mix	Mix	534	170	MAPA	10
Wang TN(19)	2004	China	EA	Children	Mix	191	129	RFLP	5
Zhai FZ(20)	2004	China	EA	Adult	Mix	64	80	PCR-RFLP	7
Bilolikar(21)	2005	UK	Mix	Children	NA	108	149	PCR-SSP	8
Gupta(22)	2005	India	SA	NA	NA	155	211	ARMS-PCR	7
Zhao HJ(23)	2005	China	EA	NA	NA	50	80	PCR-RFLP	6
Sharma(N)(5)	2006	India	SA	Adult	Atopic	248	252	Snapshot	8
Sharma(W)(5)	2006	India	SA	Adult	Atopic	240	224	Snapshot	8
Tolgyesi(24)	2006	Hungary	Caucasian	Children	Mix	144	174	PCR-RFLP	12
Kamali(25)	2007	Iran	WA	Mix	NA	203	113	Allele specific	8
								PCR	
Mak(26)	2007	China	EA	Adult	Mix	292	292	PCR-RFLP	12
Kim(27)	2008	Korea	EA	Children	Mix	715	240	PCR-RFLP	10
Kumar(28)	2008	India	SA	Mix	Mix	123	100	ARMS-PCR	8
Trajkov(29)	2008	Macedonia	Caucasian	Adult	NA	74	301	PCR-SSP	9
Aytekin(30)	2009	Turkey	WA	Children	Mix	46	67	PCR	6
Mahdaviani(31)	2009	Iran	WA	Mix	NA	27	137	PCR-SSP	8
Wang JY(32)	2009	China	EA	Children	Mix	448	510	TaqMan assay	8
Cui LY(33)	2009	China	EA	Adult	NA	100	104	PCR-RFLP	5
Dhaouadi(34)	2011	France	Caucasian	Mix	Mix	106	168	PCR-RFLP	5
Jiffri(35)	2011	Egypt	WA	Children	Mix	120	120	PCR-RFLP	8
Murk(36)	2011	USA	Mix	Children	Atopic	100	487	TaqMan assay	10
Jia N(37)	2012	China	EA	Children	NA	91	89	PCR-RFLP	8
Zheng(38)	2012	China	EA	Children	NA	198	110	PCR-RFLP	6
Li Ying(39)	2013	China	EA	Adult	NA	65	50	PCR-RFLP	6
Shaker(40)	2013	Egypt	WA	Children	Mix	100	100	PCR-RFLP	7

Abbreviations: ARMS, amplification refractory mutation system; EA, East Asian; MAPA, multiplex automated primer extension analysis; NA, not available; PCR, polymerase chain reaction; RFLP, restriction fragment length polymorphism; SA, South Asian; SSP, sequence-specific primers; WA, West Asian;

**Table 2:** Distribution of TNF-α genotype among patients and controls.

Studies	Asthma			Control			HWE
	GG	GA	AA	GG	GA	AA	p value
Louis	64	31	0	69	27	2	0.732
Winchester(C)	9	9	2	283	116	17	0.249
Winchester(A)	4	2	0	239	33	3	0.139
Buckova	102	46	3	116	38	1	0.259
Witte	164	67	4	212	55	6	0.288
Gao JM	47	52	26	44	41	11	0.759
Beghe	108	33	1	36	8	1	0.503
Guo YL	4	28	16	7	11	3	0.690
Liu DF	98	15	0	104	22	0	0.283
Shin	482	50	2	131	37	2	0.733
Wang TN	140	49	2	111	18	0	0.394
Zhai FZ	44	14	6	67	12	1	0.587
Bilolikar	50	51	7	94	46	9	0.301
Gupta	116	36	3	178	32	1	0.731
Zhao HJ	45	5	0	71	9	0	0.594
Sharma (N)	189	58	1	217	33	2	0.552
Sharma (W)	186	53	1	190	32	2	0.617
Tolgyesi	99	41	4	122	47	5	0.854
Kamali	175	28	0	103	9	1	0.137
Mak	244	47	1	250	40	2	0.774
Kim	614	95	6	219	21	0	0.479
Kumar	86	35	2	82	18	0	0.323
Trajkov	64	9	1	231	66	4	0.770
Aytekin	35	11	0	51	16	0	0.267
Mahdaviani	10	17	0	98	39	0	0.052
Wang JY	345	100	3	409	94	7	0.549
Cui LY	92	6	2	89	13	2	0.088
Dhaouadi	69	31	6	122	42	4	0.865
Jiffri	87	33	0	105	15	0	0.465
Murk	78	20	2	359	113	15	0.103
Jia N	76	14	1	82	7	0	0.699
Zheng	168	25	5	93	17	0	0.380
Li ying	45	16	4	43	6	1	0.191
Shaker	32	60	8	66	30	4	0.800

Abbreviations: HWE, Hardy-Weinberg equilibrium.

### Quantitative Data Synthesis

**Figure 2:**
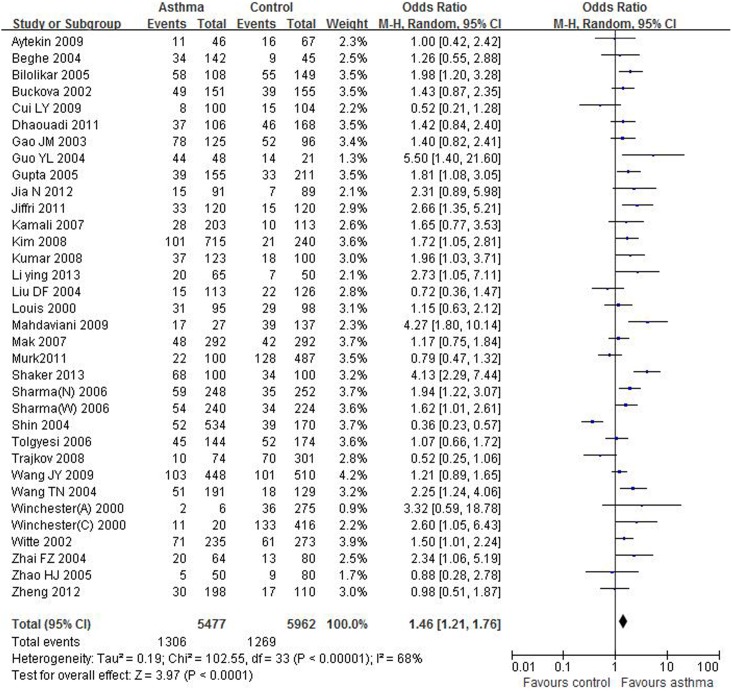
Meta-analysis for the association between asthma risk and the TNF-α rs1800629 polymorphism.

In the present study, the sample sizes for case and control groups were 5477 and 5962, respectively. A summary of the meta-analysis findings concerning association between TNF-α rs1800629 polymorphism and asthma was provided in Table 3. We found that the A allele was associated with increased asthmatic risk in overall population (OR = 0.72, 95% CI = 0.61–0.84, P<0.0001). In addition, the estimated OR1, OR2 and OR3 were 0.60 (P = 0.001), 0.88 (p = 0.41), 0.70 (P = 0.0002). These estimates suggested a recessive model, therefore GA and AA were combined and compared with GG. The pooled OR was 1.46 (95% CI 1.21–1.76, P<0.0001), demonstrating a significant association between TNF-α rs1800629 polymorphism and asthma (Figure 2). In the subgroup analysis by age, significantly increased risks were found both in adults and children with asthma (OR = 1.43, 95 CI% = 1.07–1.91, P = 0.02; OR = 1.57, 95% CI = 1.19–2.06, P = 0.001) (Table 3, Figure S1). Stratification by atopic status indicated that the rs1800629 polymorphism was significantly associated with risk of atopic asthma (OR = 1.51, 95% CI = 1.24–1.83, P<0.0001). However, no significant association was found on non-atopic asthma (Table 3, Figure S2). When stratified analysis based on ethnicity, a statistically significant association was found in West Asians and South Asians (OR = 2.47, 95% CI = 1.48–4.12, P = 0.0005; OR = 1.83, 95% CI = 1.42–2.36, P<0.00001), but not in East Asians and Caucasians (Table 3, Figure S3). Notably, heterogeneity was significantly decreased when stratified analysis was performed by atopic status (Table 3).

**Table 3:** Summary odds ratios for relationship between the TNF-αr s1800629polymorphism and asthma risk.

		No. of study	sample size	Hypothesis tests				Heterogeneity tests	
Polymorphisms	study		cases/controls	OR(95% CI)	*Z*	*p* Value	Model	*I^2^(%)*	*p* Value
G vs. A	Overall	34	10954/11924	0.72(0.61–0.84)	4.07	<0.0001	R	65	<0.00001
GG vs. AA(OR1)	Overall	34	4290/4799	0.60(0.45–0.82)	3.29	0.001	F	13	0.26
GA vs. AA(OR2)	Overall	34	1306/1269	0.88(0.65–1.20)	0.82	0.41	F	0	0.83
GG vs. GA(OR3)	Overall	34	5358/5856	0.70(0.58–0.84)	3.77	0.0002	R	66	<0.00001
GA+AA vs. GG	Overall	34	5477/5962	1.46(1.21–1.76)	3.97	<0.0001	R	68	<0.00001
GA+AA vs. GG	Adult	11	1436/1544	1.43(1.07–1.91)	2.38	0.02	R	55	0.01
GA+AA vs. GG	Children	14	2400/2992	1.57(1.19–2.06)	3.2	0.001	R	64	0.0005
GA+AA vs. GG	Atopic	7	1587/1628	1.51(1.24–1.83)	4.18	<0.0001	F	26	0.23
GA+AA vs. GG	Non-atopic	3	259/510	1.23(0.79–1.91)	0.89	0.37	F	32	0.23
GA+AA vs. GG	Caucasians	7	723/1357	1.18(0.95–1.48)	1.48	0.14	F	35	0.16
GA+AA vs. GG	Asians	24	4302/3696	1.58(1.23–2.03)	3.57	0.0004	R	73	<0.00001
GA+AA vs. GG	EA	14	3034/2097	1.27(0.91–1.77)	1.4	0.16	R	74	<0.00001
GA+AA vs. GG	WA	5	496/537	2.47(1.48–4.12)	3.46	0.0005	R	58	0.05
GA+AA vs. GG	SA	5	772/1062	1.83(1.42–2.36)	4.68	<0.00001	F	0	0.94

Abbreviations: EA, East Asian; F, fixed-effect model; OR, odds ratio; R, random-effect model; SA, South Asian; vs, versus; WA, West Asian.

### Sensitivity Analysis and Publication Bias

**Figure 3:**
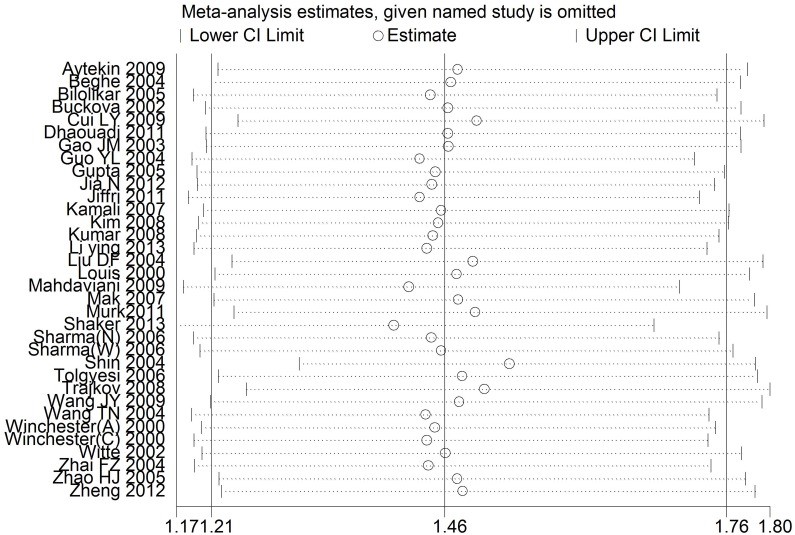
One-way sensitivity analysis for the TNF-α rs1800629 polymorphism with asthma risk.

**Figure 4:**
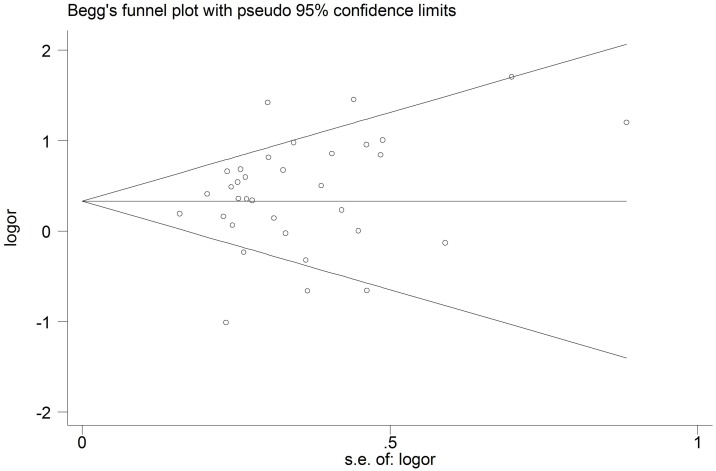
Begg’s funnel plot for publication bias on asthma risk and the TNF-α rs1800629 polymorphism.

To assess the stability of the results of this meta-analysis, sensitivity analysis was performed by sequentially excluding each study. As shown in Figure 3, sensitivity analyses indicated that the three independent studies by Shin et al. [18], Shaker et al. [39] and Trajkov et al. [29] were the main origin of the heterogeneity in the overall comparisons. The heterogeneity was obviously decreased after exclusion of these three studies (G vs. A: OR = 0.69, 95%CI = 0.61–0.78, P<0.00001, *I^2^* = 35%; GA+AA vs. GG: OR = 1.51, 95%CI = 1.31–1.73, P<0.00001, *I^2^* = 37%). Meanwhile, similar result was obtained when any single study was omitted (Figure 3). Furthermore, the pooled ORs were not significantly altered after omitting the studies which the sample size was less than 50 cases in each group (OR = 1.38, 95%CI = 1.13–1.67, P<0.001), indicating that the results of this meta-analysis were relatively stable. The shape of the funnel plot seemed symmetrical (Figure 4), and neither Egger’s test nor Begg’s test indicated any significant publication bias (P = 0.191 and 0.286, respectively).

## Discussion

Asthma is a complex inflammatory disease involving the critical actions of several important cytokines. The proinflammatory cytokines, such as TNF-α, are found in airways and are known to induce inflammatory responses and regulate immunity. TNF-α has important biological effects on airway inflammation and remodeling. Several studies have shown that high serum TNF-α level is linked to hyperresponsiveness in asthma [5], [52]. Furthermore, the concentration of TNF-α was found to be elevated in asthmatic airways and sputum [5], [53]. Because secretion of cytokine is genetically regulated at the level of transcription, and the linkage of TNF-α polymorphisms with the genotype of asthma has been demonstrated in accumulating studies. TNF-α rs1800629 polymorphism was suggested to have a significant functional effect. The A allele of this polymorphism can lead to high binding affinity of nuclear factors to the TNF promoter, resulting in a high level of transcription activity and secretion levels of TNF-α [5], [54], [55].

In harmony with those reports, a significant association was also noted for the TNF-α rs1800629 polymorphism in the overall populations in the present meta-analysis. This result suggested that individuals with the A allele (AG or AA) had 46% increased asthma risk compared to those individuals with the GG carriers. In the stratified analysis by ethnicity, significant associations were showed in Asians, but not Caucasians. This result was consistent with previous meta-analysis performed by Zhang et al. [43]. However, when we further evaluated the Asians, the positive association was shown in West Asians and South Asians, but not in East Asians. It is possible that different genetic backgrounds and environmental exposure may account for these differences. However, it should be noted that high heterogeneity was observed in East Asians group and this may distort the result.

When subgroup analysis was performed according to atopic status, significant association was observed between this polymorphism and atopic asthma risk, suggesting that the TNF-α rs1800629 polymorphism may play a role in the etiology of atopic asthma. However, there were only three studies performed with non-atopic asthma, thus the positive association still could not be excluded because studies with small sample size may have insufficient statistical power to detect a slight association. These issues should be investigated in the future studies.

Although moderate heterogeneity for this polymorphism was detected in overall analysis, results from one-way sensitivity analysis suggested high stability and reliability of our results. After subgroup by atopic status and ethnicity, the heterogeneity was effectively decreased or disappeared from atopic group, non-atopic group, Caucasians group and South Asians group, suggesting the main source of heterogeneity may be atopic status and ethnicity. Moreover, we performed funnel plots and Egger’s tests to evaluate the publication bias. The results indicated that there was no publication bias in our study.

Genome-wide association study (GWAS) is also an effective tool for the identification of genetic variants in many multi-pathogenetic diseases. Up to now, GWAS studies have successfully identified susceptibility genes for asthma by using large scale population cohorts and replication approaches [56]–[66]. Despite the fact that more than ten GWAS of asthma have been reported, there is still no direct evidence proved the association with the TNF-α region. In the study of Michel S et al, TNF-α (only one SNP, detailed data not shown) was captured indirectly by GWAS genotyping. Using the combination of GWAS genotyping data, re-genotyping and imputation, they reported that TNF (rs1800629) was associated with asthma at a nominal significance level (0.035<p≤0.05) [67]. However, GWAS studies also have deficiencies. At the first step of GWAS, significant SNP are screened in the whole genome among a small scale population in order to reduce the cost, for that it couldn’t have enough capability to discover all SNPs associated with a disease. So we supposed that some SNPs of TNF-α region derived from GWAS may be potentially associated with risk of asthma, expanding the screen criteria at the first step of GWAS may improve the power of test [68], to generate more SNPs associated with asthma, possibly including TNF-α rs1800629.

There are several limitations should be taken into account when interpreting our results. First, the numbers of published studies were limited for a comprehensive analysis, especially for the non-atopic asthmatics. Second, some studies were excluded from the study because of not providing the original genotype numbers or frequencies, which may result in selection bias. Third, our results were on the basis of unadjusted estimates. The assessment of the gene-gene and gene-environment interactions for asthma may be imprecise on account of failing to get the original data of the eligible studies. Forth, ethnicity in most studies were limited to Asians and Caucasians, the information from other ethnic groups still need further investigation.

In conclusion, this meta-analysis indicated that the TNF-α-308G/A polymorphism is significant associated with asthma. Well-designed studies with larger scale and various ethnicities are required to further confirm these associations.

## Supporting Information

Figure S1 **Subgroup analysis by age for the association between asthma risk and the TNF-α rs1800629 polymorphism (GA+AA vs. GG).**
(TIF)

Figure S2 **Subgroup analysis by atopic status for the association between asthma risk and the TNF-α rs1800629 polymorphism (GA+AA vs. GG).**
(TIF)

Figure S3 Subgroup analysis by ethnicity for the association between asthma risk and the TNF-α rs1800629 polymorphism (GA+AA vs. GG).* EA, East Asian; SA, South Asian; WA, West Asian.
(TIF)

Table S1 Scale for quality assessment of molecular association studies of asthma.
(DOC)

Checklist S1 PRISMA Checklist of items to include when reporting a systematic review or meta-analysis.
(DOC)
