# Supplementary figures and images for: Association between Tumor Necrosis Factor-α rs1800629 Polymorphism and Risk of Asthma: A Meta-Analysis
Source: PLoS One. 2014 Jun 17;9(6):e99962. doi: 10.1371/journal.pone.0099962 (PMC4061054; doi:10.1371/journal.pone.0099962)

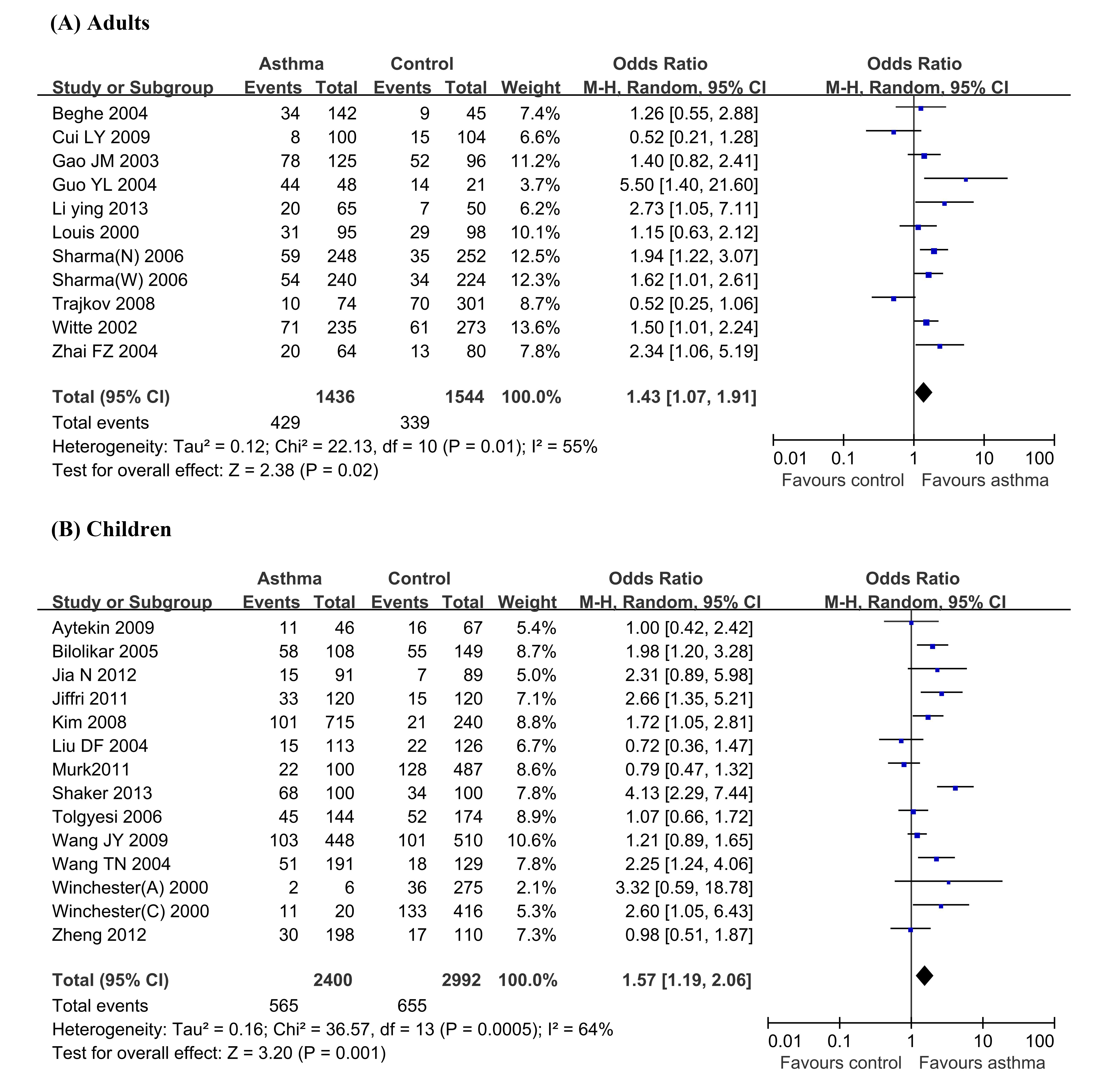

Supplement: Figure S1 — Subgroup analysis by age for the association between asthma risk and the TNF-α rs1800629 polymorphism (GA+AA vs. GG). (TIF) [file pone.0099962.s001.tif]

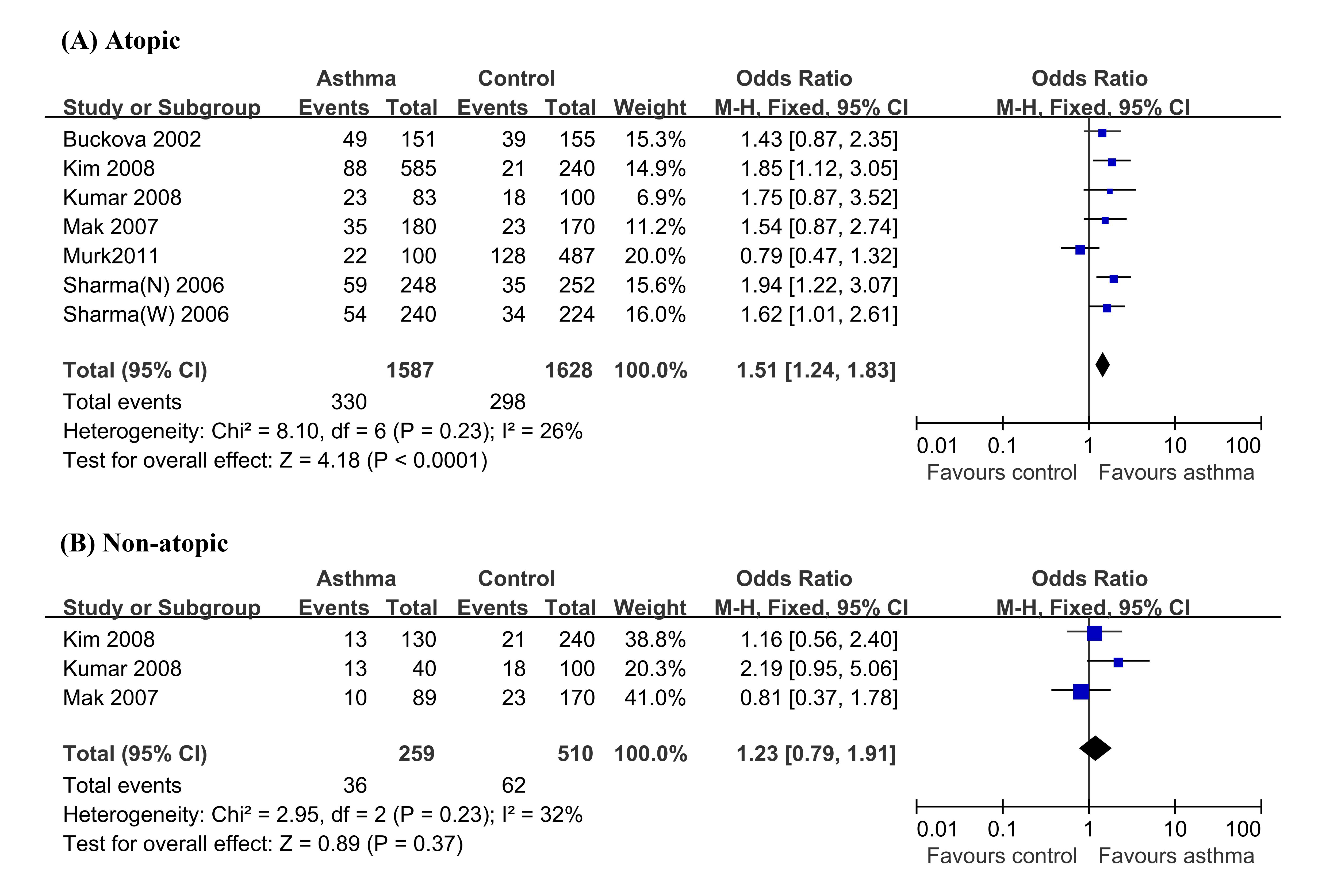

Supplement: Figure S2 — Subgroup analysis by atopic status for the association between asthma risk and the TNF-α rs1800629 polymorphism (GA+AA vs. GG). (TIF) [file pone.0099962.s002.tif]

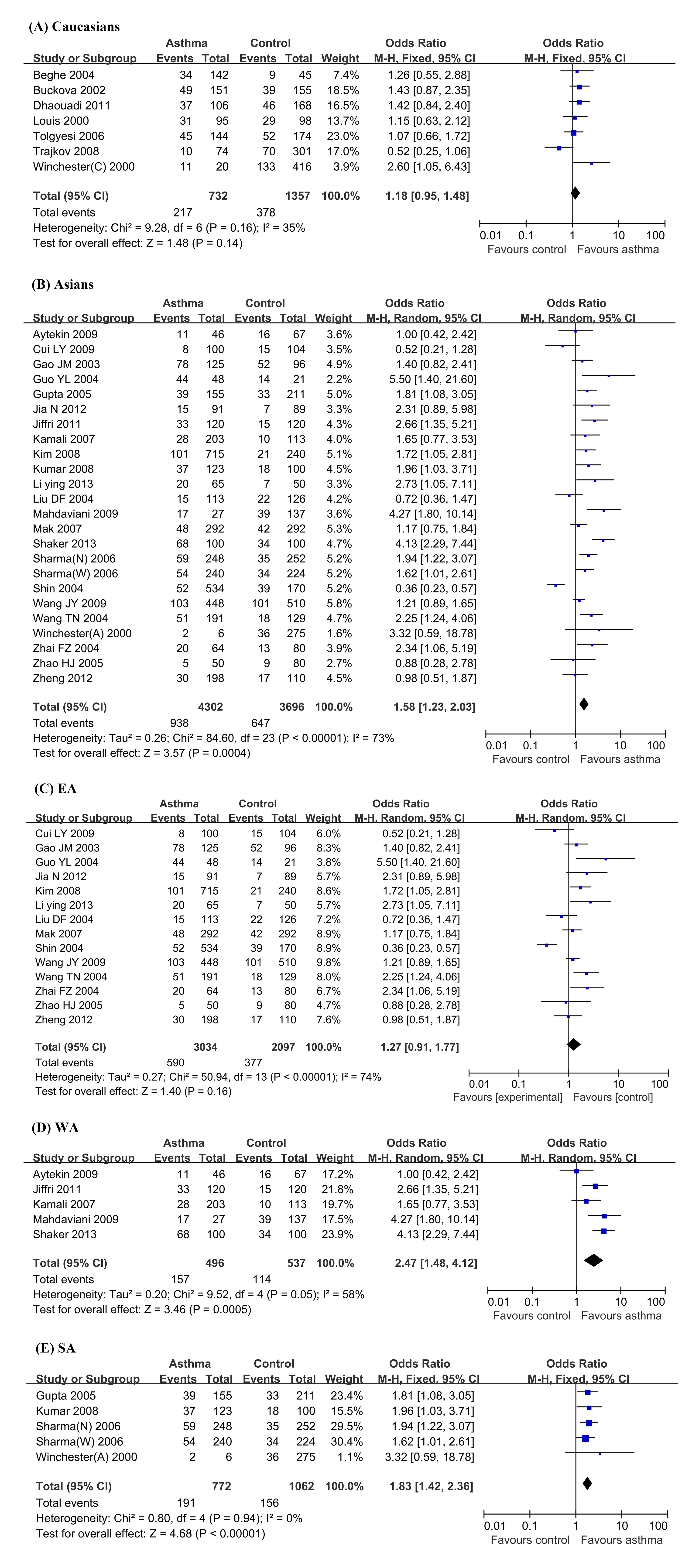

Supplement: Figure S3 — Subgroup analysis by ethnicity for the association between asthma risk and the TNF-α rs1800629 polymorphism (GA+AA vs. GG).* EA, East Asian; SA, South Asian; WA, West Asian. (TIF) [file pone.0099962.s003.tif]
