# Supplementary material for: Association between Tumor Necrosis Factor-α rs1800629 Polymorphism and Risk of Asthma: A Meta-Analysis
Source: PLoS One. 2014 Jun 17;9(6):e99962. doi: 10.1371/journal.pone.0099962 (PMC4061054; doi:10.1371/journal.pone.0099962)
Supplement: Table S1 — Scale for quality assessment of molecular association studies of asthma. (DOC) [file pone.0099962.s004.doc]

Table S1. Scale for quality assessment of molecular association studies of asthma

| **Criteria** | **Score** |
| --- | --- |
| **Representativeness of cases** |  |
| Consecutive/randomly selected from case population with clearly deﬁned sampling frame | 2 |
| Consecutive/randomly selected from case population without clearly deﬁned sampling frame or with extensive inclusion/exclusion criteria | 1 |
| No method of selection described | 0 |
| **Representativeness of controls** |  |
| Controls were consecutive/randomly drawn from the same sampling frame (ward/community) as cases | 2 |
| Controls were consecutive/randomly drawn from a different sampling frame as cases | 1 |
| Not described | 0 |
| **Ascertainment of asthma** |  |
| Clearly described objective criteria for diagnosis of asthma | 2 |
| Diagnosis of asthma by patient self-report or by patient history | 1 |
| Not described | 0 |
| **Ascertainment of controls** |  |
| Controls were tested to screen out asthma,i.e., measured FEV 1 * or PEFR* | 2 |
| Controls were subjects who did not report asthma;no objective testing | 1 |
| Not described | 0 |
| **Genotyping examination** |  |
| Genotyping done under ‘‘blinded’’ condition | 1 |
| Unblinded or not mentioned | 0 |
| **Hardy-Weinberg equilibrium** |  |
| Hardy-Weinberg equilibrium in control group | 2 |
| Hardy-Weinberg disequilibrium in control group | 1 |
| No checking for Hardy-Weinberg equilibrium | 0 |
| **Association assessment** |  |
| Assess association between genotypes and asthma with appropriate statistics and adjustment for confounders | 2 |
| Assess association between genotypes and asthma with appropriate statistics without adjustment for confounders | 1 |
| Inappropriate statistics used | 0 |
| **Response rate** |  |
| Response rates for both groups are the same,i.e., to within 5% | 2 |
| Response rates are different, between 5% and 10% | 1 |
| Response rates are more than 10% different, or no mention of response rates | 0 |

* FEV1, forced expiratory volume in 1 second; PEFR, peak expiratory ﬂow rate.
